# Perioperative nursing management in video-assisted retroperitoneal debridement for severe acute pancreatitis with infected peripancreatic necrosis: a retrospective single-center case series of 41 patients

**DOI:** 10.3389/fsurg.2026.1839246

**Published:** 2026-06-11

**Authors:** Huali Zhang, Dongyuan Chen, Hefeng Tian, Xin Xu

**Affiliations:** Nursing Department, Sir Run Run Shaw Hospital, Zhejiang University School of Medicine, Hangzhou, Zhejiang, China

**Keywords:** infected pancreatic necrosis, minimally invasive surgery, perioperative nursing, severe acute pancreatitis, video-assisted retroperitoneal debridement

## Abstract

**Background:**

Severe acute pancreatitis (SAP) complicated by infected peripancreatic necrosis (IPN) represents a life-threatening condition. Video-assisted retroperitoneal debridement (VARD) is a preferred minimally invasive surgical approach for managing this disease; however, detailed reports on the perioperative nursing care for this procedure remain limited. This study summarizes the nursing experience and management strategies in 41 patients who underwent VARD at our institution.

**Materials and methods:**

We conducted a retrospective, single-center, uncontrolled case series of 41 consecutive SAP patients with IPN who underwent VARD between July 2024 and June 2025. We implemented a standardized perioperative nursing protocol that included the following components: 1) preoperative preparation (multidisciplinary team, patient assessment, organ support, multimodal analgesia, emergency plan); 2) intraoperative nursing care (instruments coordination, VARD assistance, positioning, sterile field); and 3) postoperative management (individualized drainage, fluid therapy, stepwise nutrition, complication prevention). Patient characteristics, surgical and nursing-sensitive outcomes were recorded.

**Results:**

The cohort comprised 30 males and 11 females, with a mean age of 52.9 ± 10.6 years. The mean operative time was 133.9 ± 26.1 min, with mean blood loss 92.0 ± 37.6 mL and no intraoperative transfusions. Six patients (14.6%; 95% CI 6.9%–28.1%) required a second debridement, of whom two (4.9%; 95% CI 1.3%–16.7%) underwent a third procedure. Postoperative intra-abdominal hemorrhage occurred in one patient (2.4%; 95% CI 0.4%–14.2%). No new-onset organ failure or mortality occurred. The mean lengths of ICU stay and hospital stay were 10.1 ± 4.4 days (median 9.0 days, IQR 7.0–11.0) and 34.9 ± 7.1 days (median 34.0 days, IQR 29.0–39.5), respectively. Nursing-sensitive complications occurred at low rates in this cohort, including no pressure injuries, and low incidences of unplanned drain dislodgement, Catheter-related bloodstream infection (CRBSI), and Catheter-associated urinary tract infection (CAUTI).

**Conclusions:**

In this retrospective, single-center, uncontrolled case series, the implemented perioperative nursing protocol was feasible and associated with acceptable perioperative outcomes, with low rates of nursing-sensitive complications. Owing to the study design, causal conclusions cannot be drawn. Future prospective, controlled studies are needed to validate these findings.

## 1 Introduction

Severe acute pancreatitis (SAP) is a common and clinically challenging abdominal emergency, characterized by an unpredictable clinical trajectory and high associated mortality (1). It is estimated that 40%–70% of patients progress to infected pancreatic necrosis (IPN) during the later stages of the disease. Severe secondary infection of necrotic tissue within the pancreas and retroperitoneum marks the pivotal point in the pathologic process of SAP and can cause a precipitous deterioration and increased mortality.

Open necrosectomy can achieve source control but entails major surgical trauma, significant morbidity, and prolonged recovery. Over the past decade, minimally invasive techniques have become increasingly recognized as viable alternatives (2, 3). Among them, video-assisted retroperitoneal debridement (VARD) is considered a valuable component of the minimally invasive step-up approach for selected patients with IPN. It provides direct access to infected necrotic collections without entering the peritoneal cavity, reducing surgical trauma and recovery, possibly (4).

Although VARD for IPN has gained increasing use, there is very little in the literature about detailed descriptions of perioperative nursing care for this technique. Nursing care is a key factor that influence procedural success and postoperative patient recovery, especially in complex, critically ill populations. We retrospectively reviewed our institutional experience with 41 consecutive patients who underwent this procedure for SAP complicated by IPN from July 2024 to June 2025. Herein, we clearly and methodically present the nursing management strategies used in the preoperative, intraoperative, and postoperative phases that contributed to the favorable clinical outcomes in this series.

## 2 Materials and methods

### 2.1 Study design and patients

This was a retrospective, single-center, uncontrolled case series involving 41 consecutive SAP patients complicated by IPN who underwent VARD between July 2024 and June 2025. As a tertiary referral center, our hospital receives a large number of patients with IPN from referring hospitals. The perioperative nursing protocol described below was developed from published guidelines, multidisciplinary team consensus, and iterative institutional refinement over time.

Using procedure codes for VARD, we identified all consecutive patients who underwent this procedure through the hospital's surgical database during the study period. We could not generate a patient flow diagram because our database search was procedure-based and therefore did not capture all SAP/IPN patients who were managed conservatively or with PCD alone during the same period. All cases fulfilled the diagnostic criteria for SAP as outlined in the 2025 Guidelines on Acute Pancreatitis from the International Association of Pancreatology (5). IPN was diagnosed based on contrast-enhanced computed tomography (CT) findings of gas bubbles within necrotic collections and/or positive culture from percutaneous aspiration. More importantly, all patients had a Marshall score of 2 or higher, confirming organ dysfunction, and none responded adequately to maximal medical therapy and percutaneous drainage for sepsis, therefore all ultimately required surgical intervention.

Notably, the majority of patients (*n* = 31, 75.6%) were transferred from other hospitals, often already with PCD catheters in place. Consequently, the exact time from symptom onset to PCD or to VARD could not be reliably determined from the available records.

### 2.2 Study endpoints

The primary endpoints were the feasibility of the perioperative nursing protocol (defined as successful completion of the protocol without major deviation) and the incidence of nursing-sensitive outcomes (pressure injuries, unplanned tube dislodgement, catheter-related bloodstream infection, and catheter-associated urinary tract infection). Secondary endpoints included operative time, estimated blood loss, repeat debridement rate, postoperative hemorrhage, ICU stay, hospital stay, and mortality.

### 2.3 Surgical technique

The procedure began with the patient placed in a left lateral decubitus position at a 90° angle, and the lumbar bridge was elevated accordingly. A 2 cm incision was created either along the pre-existing catheter tract or at an optimal entry point predetermined by imaging. A two- or three-port technique was adopted, with port configuration tailored to the distribution of retroperitoneal necrosis. To establish an adequate working space, superficial necrotic tissue was initially evacuated using oval forceps, followed by insertion of a 12 mm trocar. In select cases, a visualized trocar was employed for direct puncture into the retroperitoneal abscess cavity. The working space was subsequently expanded by insufflation. Under laparoscopic visualization, dissection proceeded along the pararenal space, extending inferiorly toward the pelvis and superiorly toward the pancreatic tail. Debridement was performed using a systematic “three-step method” (oval forceps–laparoscopic instruments–oval forceps), alternating between graspers or suction devices and oval forceps to ensure thorough removal of deep-seated infected necrotic tissue. The abscess cavity was then repeatedly irrigated with hydrogen peroxide. Hydrogen peroxide (diluted 1:3 with normal saline) was used for irrigation to physically dislodge debris and provide local antiseptic effect, following institutional protocol. No hydrogen peroxide-related complications (e.g., gas embolism, tissue damage) were seen in this series.

Thereafter, the patient was sequentially repositioned to a 90° right lateral decubitus position and subsequently to supine, allowing debridement of the right retroperitoneal space and lesser sac using laparoscopic graspers. Any vascular bleeding encountered was controlled with suture ligation under direct visualization. The same three-step debridement technique was used on the right side. Upon completion of debridement in each position, irrigation drains were placed directly into the abscess cavity under laparoscopic guidance, and dual-lumen drains were left *in situ* to facilitate continuous postoperative irrigation and drainage (6).

### 2.4 Perioperative nursing management protocol

A comprehensive, multidisciplinary nursing protocol was implemented for all patients, organized throughout the preoperative, intraoperative, and postoperative phases. This protocol is described in detail below.

#### 2.4.1 Preoperative preparation

Multidisciplinary Team Formation: A multidisciplinary team was convened, consisting of intensivists, gastroenterologists, surgical group, interventional radiologists, and anesthesiologists, and it functioned under a well-defined emergency protocol that facilitated efficient treatment planning while at the same time maximizing patient safety (7).

##### 2.4.1.1 Comprehensive preoperative assessment

Preoperative laboratory and diagnostic findings were systematically reviewed by the surgical and nursing teams to confirm the patient's readiness for the procedure (8). For non-intubated patients, nursing staff evaluated their psychological status, provided supportive interventions to alleviate anxiety, and verified compliance with preoperative fasting guidelines.

##### 2.4.1.2 Multi-Organ function support and monitoring

Comprehensive preoperative support was imperative (9). The standard approach included invasive hemodynamic monitoring, lung-protective ventilation strategies, hourly urine output measurement, strict glycemic control, and active patient warming, with routine assessment of arterial blood gases, electrolyte levels, and serum lactate to actively maintain hemodynamic stability (10).

##### 2.4.1.3 Multimodal analgesia and abdominal distension management

A multimodal analgesic approach was preferred, usually a combination of an NSAID with a regional technique (such as epidural analgesia or TAP block) to reduce opioid requirements, consistent with local institutional practice (11). Essential measures for abdominal distension included maintaining effective nasogastric decompression with close monitoring of output, along with serial measurement of intra-abdominal pressure (IAP) for early identification of intra-abdominal hypertension.

##### 2.4.1.4 Surgical equipment and supplies preparation

Prior to surgery, the circulating nurse ensured all equipment (imaging system, insufflator, electrosurgical unit) was functional. The scrub nurse prepared single-use items such as trocars, catheters, retrieval bags, and laparoscopic instruments.

##### 2.4.1.5 Emergency protocol development

An intraoperative bleeding protocol was established. In cases of hemodynamic instability or uncontrolled hemorrhage, the circulating nurse should mobilize additional personnel while the scrub nurse prepared hemostatic instruments.

#### 2.4.2 Intraoperative nursing care

##### 2.4.2.1 Nursing support for establishing the retroperitoneal approach

The nursing team assisted at every stage by ensuring that the necessary instruments were available in a timely manner (No. 11 blade, dissecting forceps, long-handled oval forceps, ultrasonic scalpel). Following development of the retroperitoneal space, a 10 mm trocar was introduced, and the camera connected. After placing a 12 mm trocar into the abscess cavity and initiating insufflation, a 5 mm trocar was inserted at the mid-axillary line under laparoscopic visualization to create the operative port.

##### 2.4.2.2 Coordination of necrotic tissue debridement

**Figure 1:**
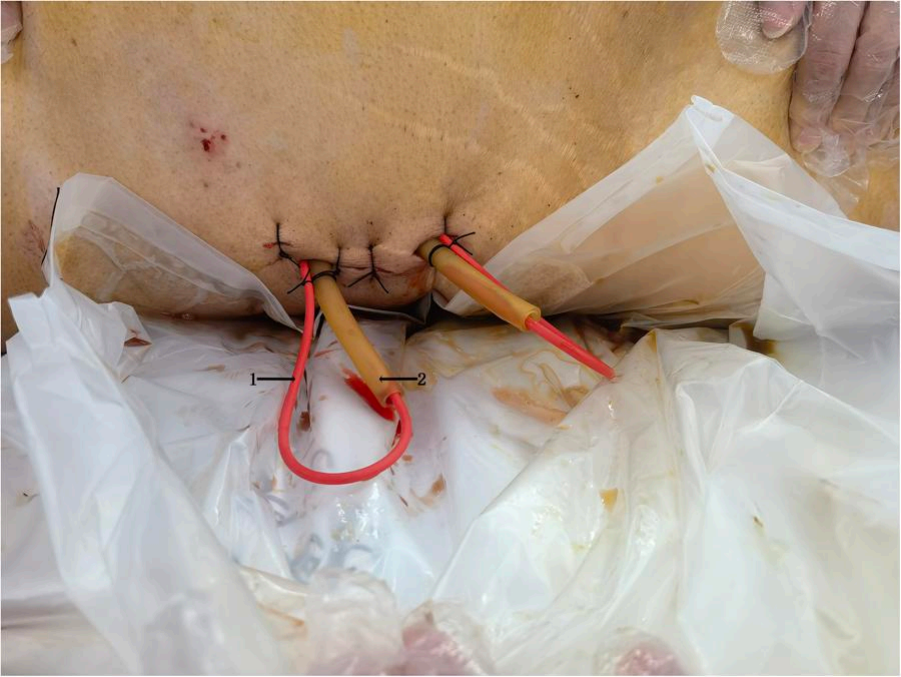
Two drainage devices placed in a staggered arrangement to prevent cross-contamination. Arrow 1 points to the red irrigation tube used for postoperative abscess irrigation; arrow 2 points to the yellow drainage tube used for postoperative drainage.

Deep infected necrotic tissue was removed using a three-step technique alternating between laparoscopic graspers, aspirators, and oval forceps (6). Instruments were passed in synchrony with the surgeon's movements. The abscess cavity was repeatedly irrigated with hydrogen peroxide, after which drainage tubes were placed and secured (Figure 1). Sterile draping was maintained at all times.

##### 2.4.2.3 Programmed positioning management

**Figure 2:**
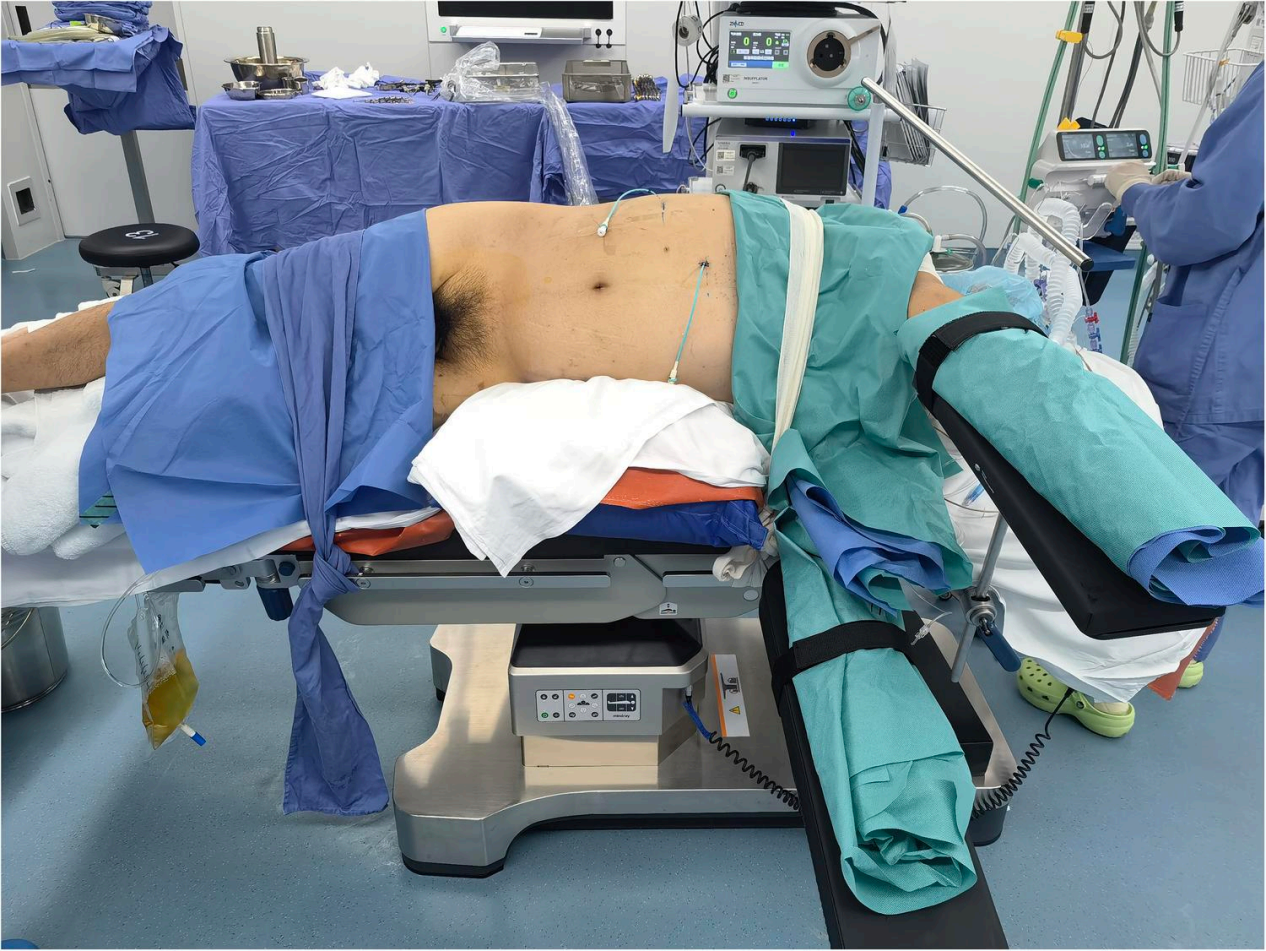
Surgical positioning and limb protection. A soft cushion is placed under the axilla to prevent brachial plexus injury, and another cushion is placed between the lower limbs to alleviate pressure, thereby reducing the risk of nerve injury and pressure ulcers.

The patient was repositioned according to the surgical sequence: first in a 90° left lateral decubitus position, then 90° right lateral decubitus, and finally supine. Silicone pads and axillary pillows were utilized to protect against brachial plexus injury (12) (Figure 2). All repositioning maneuvers were performed collaboratively by the anesthesiologist, nurse, and surgeon to avoid tension on the incision and maintain airway patency.

##### 2.4.2.4 Sterile waterproof barrier

During high-volume irrigation, we used sterile surgical film and waterproof drapes to isolate the incision and reduce the risk of contamination.

#### 2.4.3 Postoperative nursing management

##### 2.4.3.1 Systematic drainage tube management (“One drainage tube, One strategy”)

Postoperative management of drainage tubes was individualized. Each tube was clearly labeled with its name, insertion date, and purpose. Care protocols varied by tube type - retroperitoneal drains, nasogastric tubes, or nasojejunal tubes - and a daily multidisciplinary review helped decide the appropriate timing for removal.

##### 2.4.3.2 Precision fluid management and maintenance of internal homeostasis

Fluid therapy was adjusted dynamically based on serial assessments. Invasive blood pressure (IBP) and central venous pressure (CVP) monitoring were routinely employed to assess hemodynamic status. In selected cases, pulse index continuous cardiac output (PICCO) monitoring provided additional real-time data (13, 14). During the initial 24 h, crystalloids were infused at rates titrated to CVP, IBP, and urine output (14). From 24 to 72 h, attention shifted to achieving fluid balance and correcting electrolyte disturbances (15). After 72 h, once hemodynamic stability was achieved, a negative balance was gradually introduced.

##### 2.4.3.3 Stepwise nutritional support

During the initial 48 h, parenteral nutrition (PN) was provided, as most patients had hemodynamic instability or abdominal compartment concerns that precluded early enteral nutrition (EN) (16). Between 48 h and 7 days, once bowel sounds and flatus had returned and hemodynamic stability was restored, EN was introduced using a short-chain peptide formula. After the first week, patients tolerating full-strength EN were switched to a whole-protein formula, and oral intake was gradually introduced (17). This step-up approach (PN first, then EN) reflects our institutional practice for this critically ill cohort and is not intended to conflict with general guidelines favoring early EN when feasible.

##### 2.4.3.4 Complication prevention and monitoring

Postoperative care was systematically structured to prevent intra-abdominal hemorrhage, pancreatic fistula, and infection (18), with evidence-based interventions tailored to each complication. To reduce bleeding risk, patients remained supine for the first six hours, with all drainage tubes securely fixed. For pancreatic fistula prevention, we employed early initiation of fasting, administration of somatostatin analogues, and maintenance of drain patency. Infection prevention measures emphasized respiratory care: deep breathing exercises, regular repositioning, back percussion, and nebulized treatments.

**Figure 3:**
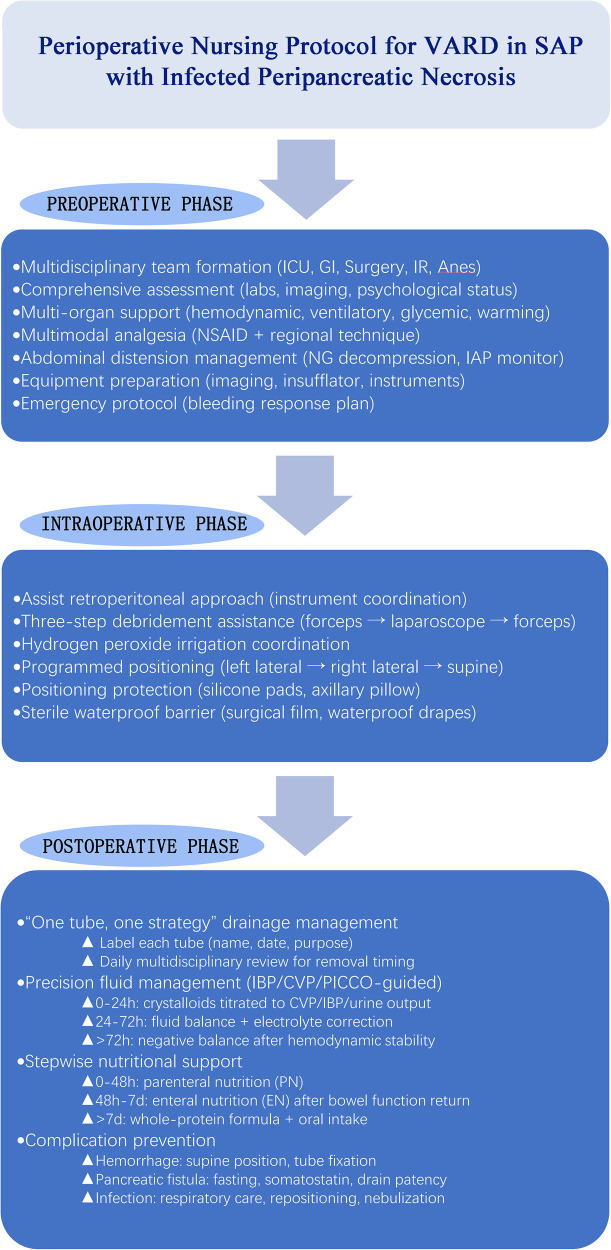
Flowchart of the perioperative nursing protocol for patients with severe acute pancreatitis and infected peripancreatic necrosis undergoing video-assisted retroperitoneal debridement (VARD). The protocol is organized into three phases: preoperative (MDT formation, assessment, organ support, analgesia, equipment, emergency protocol), intraoperative (approach assistance, three-step debridement, irrigation, programmed positioning with protection, sterile barrier), and postoperative (individualized drainage care, precision fluid management, stepwise nutritional support, and complication prevention). MDT, multidisciplinary team; IBP, invasive blood pressure; CVP, central venous pressure; PICCO, pulse index continuous cardiac output; PN, parenteral nutrition; EN, enteral nutrition.

The complete protocol is summarized in Figure 3.

### 2.5 Statistical analysis

Due to the descriptive nature of this case series, no hypothesis testing was performed. Continuous variables are presented as mean ± standard deviation (SD). For skewed variables (e.g., ICU stay, hospital stay), medians and interquartile ranges (IQR) are also reported where indicated. Categorical variables are presented as frequencies and percentages. All data were analyzed using SPSS version 26.0 (IBM Corp., Armonk, NY, USA). No statistician was involved in the analysis, as the study was purely descriptive.

## 3 Results

### 3.1 Patient characteristics

A total of 41 patients were included in this study. The cohort comprised 30 males and 11 females, with a mean age of 52.9 ± 10.6 years. The etiologies of pancreatitis were gallstones (*n* = 28, 68.3%), hypertriglyceridemia (*n* = 8, 19.5%), trauma (*n* = 3, 7.3%), and alcohol (*n* = 2, 4.9%). The most common comorbidities were hypertension (*n* = 17, 41.5%), diabetes mellitus (*n* = 14, 34.1%), and coronary atherosclerosis (*n* = 5, 12.2%). All 41 patients had positive preoperative cultures. Specimens were obtained from abscess aspirate/necrotic tissue (*n* = 37 positive cultures), drain fluid (*n* = 32), and blood (*n* = 11); a single patient could contribute multiple positive cultures from different sources. The most common organisms were Escherichia coli, Klebsiella pneumoniae, and Enterococcus species. Polymicrobial infections were identified in 31 patients. Each patient had a Marshall score of 2 or higher, indicating the presence of organ dysfunction. Despite maximal medical therapy and percutaneous drainage, all patients exhibited persistent signs of sepsis, ultimately necessitating retroperitoneal laparoscopic debridement (Table 1).

**Table 1:** Patient characteristics.

Characteristics	Value
Gender (n, %)	
Male	30
Female	11
Age	52.9 ± 10.6
BMI (kg/m^2^)	28.4 ± 7.2
Etiology (n, %)	
Gallstones	28 (68.3)
Hypertriglyceridemia	8 (19.5)
Trauma	3 (7.3)
Alcohol	2 (4.9)
Marshall Score (n, %)	
2–4	19 (46.3)
5–7	15 (36.6)
≥8	7 (17.1)
Pathogen isolates^a^ (n, %)	
Gram-negative bacteria	74 (81.3)
Gram-positive bacteria	15 (16.5)
Fungus	2 (2.2)
Comorbidities (n, %)	
Hypertension	17 (41.5)
Diabetes mellitus	14 (34.1)
Coronary atherosclerosis	5 (12.2)

a Multiple isolates could be cultured from a single patient; the table shows the total number of isolates (*N* = 91). The denominator for percentages is the total number of isolates.

### 3.2 Surgical outcomes

All 41 patients successfully underwent retroperitoneoscopic debridement. The mean operative time was 133.9 ± 26.1 min, with a mean estimated blood loss of 92.0 ± 37.6 mL; no intraoperative blood transfusions were required. Six patients (14.6%; 95% CI 6.9%–28.1%) underwent a second debridement, with two (4.9%; 95% CI 1.3%–16.7%) requiring a third procedure. One patient (2.4%; 95% CI 0.4%–14.2%) developed postoperative intra-abdominal hemorrhage, which was successfully managed with interventional embolization. No new-onset organ failure or perioperative mortality was observed. Patients had a mean ICU stay of 10.1 ± 4.4 days (median 9.0 days, IQR 7.0–11.0) and a mean hospital stay of 34.9 ± 7.1 days (median 34.0 days, IQR 29.0–39.5) (Table 2).

**Table 2:** Surgical outcomes.

Outcomes	Value
Operative time (minutes)	133.9 ± 26.1
Estimated blood loss (mL)	92.0 ± 37.6
Intraoperative blood transfusions	0
Second debridement procedure (n, %)^a^	6 (14.6; 95% CI 6.9%–28.1%)
Third debridement procedure (n, %)^a^	2 (4.9; 95% CI 1.3%–16.7%)
Postoperative hemorrhage (n, %)^a^	1 (2.4; 95% CI 0.4%–14.2%)
New-onset organ failure	0
Perioperative mortality	0
ICU stay (days)	10.1 ± 4.4 (median 9.0, IQR 7.0–11.0)
Hospital stay (days)	34.9 ± 7.1 (median 34.0, IQR 29–39.5)

a Wilson score method was used for confidence interval estimation.

### 3.3 Nursing-sensitive outcomes

From the retrospective records, we extracted several nursing-sensitive outcomes. No pressure injuries (stage ≥1) were recorded. One patient (2.4%; 95% CI 0.4%–14.2%) had an unplanned retroperitoneal drain dislodgement due to loosening of the fixation suture. Since the drain output was minimal at that time, the surgical team decided not to reinsert a new drain, and the patient recovered without further issues. Postoperative respiratory complications occurred in three patients (7.3%; 95% CI 2.5%–19.3%), including two with pneumonia and one with pleural effusion that required thoracentesis. Catheter-related bloodstream infection (CRBSI) developed in one patient (2.4%; 95% CI 0.4%–14.2%) and resolved after catheter removal and antibiotics. Catheter-associated urinary tract infection (CAUTI) occurred in two patients (4.9%; 95% CI 1.3%–16.7%), both of whom were treated with catheter replacement and antibiotic therapy. Overall, the incidence of nursing-sensitive complications was low in this cohort, though the retrospective design may have led to underreporting. Nursing-sensitive outcomes are summarized in Table 3.

**Table 3:** Nursing-sensitive outcomes.

Outcomes	N (%)^a^
Pressure injuries (stage ≥1)	0 (0)
Unplanned tube dislodgement	1 (2.4; 95% CI 0.4%–14.2%)
Respiratory complications	3 (7.3; 95% CI 2.5%–19.3%)
Catheter-related bloodstream infection (CRBSI)	1 (2.4; 95% CI 0.4%–14.2%)
Catheter-associated urinary tract infection (CAUTI)	2 (4.9; 95% CI 1.3%–16.7%)

a Wilson score method was used for confidence interval estimation.

## 4 Discussion

Severe acute pancreatitis (SAP) complicated by infected peripancreatic necrosis (IPN) is associated with a high risk of late mortality and thus presents major challenges for both medicine and nursing (19). Video-assisted retroperitoneal debridement (VARD) is widely regarded as an effective minimally invasive approach that reduces surgical trauma (4). In the current step-up management of IPN, VARD is primarily indicated for retroperitoneal or lateral necrotic collections, whereas percutaneous drainage serves as the initial drainage procedure and endoscopic necrosectomy is preferred for centrally located collections (20, 21). Nevertheless, the quality of perioperative nursing care may play a role in procedural outcomes and patient recovery.

Our findings suggest that a systematic, thorough approach to perioperative nursing may be associated with favorable outcomes in this cohort of 41 patients. Preoperatively, we focused on multidisciplinary coordination, multi-organ support, and metabolic stabilization, to optimally prepare the patient for surgery. Because a well-organized multidisciplinary team followed strict protocols, clinical problems could be recognized and addressed promptly.

Intraoperatively, the priorities were to ensure proper equipment function and maintain stable vital signs. Anticipatory nursing - anticipating the surgeon's needs and handling instruments with optimal timing - may have contributed to surgical efficiency and a relatively short operative time in this series. Equally important was our attention to patient positioning, with active protection of pressure points and smooth, coordinated repositioning, which reduced the risk of positioning-related injuries while maintaining adequate surgical exposure.

Postoperative management focused on three key elements: individualized drainage tube care following a “one tube, one strategy” approach, active complication prevention, and stepwise nutritional and rehabilitative support. Regarding nutrition, we followed a stepwise nutritional protocol, advancing from parenteral to enteral nutrition as gastrointestinal function returned - an approach consistent with current evidence supporting early enteral nutrition in SAP (17, 22). Our fluid management strategy was similarly tailored to hemodynamic parameters and the phase of resuscitation, helping to address the complex fluid shifts seen in this patient population. The low rate of intra-abdominal hemorrhage (2.4%) and the absence of new-onset organ failure or mortality in this series suggest that this nursing protocol may have contributed to the favorable outcomes observed.

Following the reviewer's suggestion, we pulled nursing-sensitive indicators from the records. No pressure injuries occurred, suggesting that our positioning protocol (silicone pads, axillary pillows, and team repositioning) worked well. The unplanned drain dislodgement rate (2.4%; 95% CI 0.4%–14.2%) was low and did not lead to adverse clinical consequences. Respiratory complications occurred in 7.3% (95% CI 2.5%–19.3%) of patients, reflecting the critical illness of this population; these were managed with standard intensive care measures. The rates of CRBSI (2.4%; 95% CI 0.4%–14.2%) and CAUTI (4.9%; 95% CI 1.3%–16.7%) were within the typical range for critically ill surgical patients (23). These numbers are reassuring, but firm conclusions cannot be drawn given the retrospective, single-center design. Trials with prospective nursing-sensitive data are needed in the future.

The nursing protocol described in this report was developed and used in a tertiary referral center with a dedicated multidisciplinary team, experienced VARD surgeons, and a nurse-to-patient ratio that supports intensive perioperative monitoring. Reproducibility in other settings would require: (1) the center's familiarity with retroperitoneal minimally invasive surgery; (2) access to interventional radiology and critical care; and (3) a nursing team trained to follow protocol-driven drainage and complication management. Centers with fewer resources or less experience may need to adjust the protocol to fit their local setting. Multi-center studies are needed to assess its reproducibility and external validity.

This study has several important limitations. First, the design is retrospective, descriptive, and from a single center without a control group, so causal conclusion cannot be drawn. We cannot determine whether the observed favorable outcomes are attributable to the nursing protocol, the surgical technique, patient selection, timing of intervention, ICU support, or the overall institutional experience. Second, the sample size is modest (*n* = 41) and derived from a single center, which limits generalizability. Our findings may not be reproducible in centers with different patient volumes, staffing levels (e.g., nurse-to-patient ratios), surgical expertise, or ICU resources. Multi-center, prospective studies with larger sample sizes are needed to validate the protocol's reproducibility and external validity. Third, due to the retrospective design, some nursing-sensitive indicators were not systematically captured. Fourth, the absence of a control group means we cannot isolate the specific contribution of the nursing protocol from other concurrent interventions. Fifth, the majority of patients were transferred from other hospitals, often with PCD catheters already in place, so reliable data on the timing from symptom onset to PCD or to VARD could not be obtained. Sixth, while we attempted to extract nursing-sensitive outcomes retrospectively, the reported rates of complications such as CRBSI and CAUTI may be underestimated due to incomplete documentation or variation in diagnostic practices. Consequently, this study is intended to describe our perioperative nursing protocol and report its feasibility and associated outcomes, not to establish causality. Prospective, controlled studies are required to determine the specific contribution of the nursing protocol and to validate these findings in diverse healthcare settings.

## 5 Conclusion

In summary, for patients with SAP complicated by IPN undergoing VARD, the perioperative nursing protocol described here was feasible and associated with acceptable outcomes. Key elements of this approach include multidisciplinary teamwork, individualized drainage tube management, and continuous, active monitoring. However, because of the retrospective, single-center, uncontrolled design, causal attribution to the nursing protocol alone is not possible. This study provides a descriptive template for standardization of perioperative nursing care in this challenging population. Before widespread adoption, the protocol should be tested in diverse healthcare settings to confirm its reproducibility. Future prospective, controlled studies are needed to validate these findings.

## Data Availability

The raw data supporting the conclusions of this article will be made available by the authors, without undue reservation.
